# Comparative Genomic and Metabolomic Analysis of *Termitomyces* Species Provides Insights into the Terpenome of the Fungal Cultivar and the Characteristic Odor of the Fungus Garden of *Macrotermes natalensis* Termites

**DOI:** 10.1128/msystems.01214-21

**Published:** 2022-01-11

**Authors:** Nina B. Kreuzenbeck, Elena Seibel, Jan W. Schwitalla, Janis Fricke, Benjamin H. Conlon, Suzanne Schmidt, Almuth Hammerbacher, Tobias G. Köllner, Michael Poulsen, Dirk Hoffmeister, Christine Beemelmanns

**Affiliations:** a Group of Chemical Biology of Microbe-Host Interactions, Leibniz Institute for Natural Product Research and Infection Biology–Hans-Knöll Institute (HKI), Jena, Germany; b Section for Ecology and Evolution, Department of Biology, University of Copenhagen, Copenhagen, Denmark; c Department of Zoology and Entomology, Forestry and Agricultural Biotechnology Institute (FABI), University of Pretoria, Pretoria, South Africa; d Max Planck Institute for Chemical Ecology, Department of Biochemistry, Jena, Germany; e Associated Group of Pharmaceutical Microbiology, Leibniz Institute for Natural Product Research and Infection Biology–Hans-Knöll Institute (HKI), Jena, Germany; f Friedrich-Schiller-Universität Jena, Department of Pharmaceutical Biology at the Hans-Knöll Institute, Jena, Germany; University of Sao Paulo

**Keywords:** *Termitomyces*, secondary metabolism, symbiosis, terpenes

## Abstract

Macrotermitinae termites have domesticated fungi of the genus *Termitomyces* as food for their colony, analogously to human farmers growing crops. Termites propagate the fungus by continuously blending foraged and predigested plant material with fungal mycelium and spores (fungus comb) within designated subterranean chambers. To test the hypothesis that the obligate fungal symbiont emits specific volatiles (odor) to orchestrate its life cycle and symbiotic relations, we determined the typical volatile emission of fungus comb biomass and *Termitomyces* nodules, revealing α-pinene, camphene, and d-limonene as the most abundant terpenes. Genome mining of *Termitomyces* followed by gene expression studies and phylogenetic analysis of putative enzymes related to secondary metabolite production encoded by the genomes uncovered a conserved and specific biosynthetic repertoire across strains. Finally, we proved by heterologous expression and *in vitro* enzymatic assays that a highly expressed gene sequence encodes a rare bifunctional mono-/sesquiterpene cyclase able to produce the abundant comb volatiles camphene and d-limonene.

**IMPORTANCE** The symbiosis between macrotermitinae termites and *Termitomyces* is obligate for both partners and is one of the most important contributors to biomass conversion in the Old World tropic’s ecosystems. To date, research efforts have dominantly focused on acquiring a better understanding of the degradative capabilities of *Termitomyces* to sustain the obligate nutritional symbiosis, but our knowledge of the small-molecule repertoire of the fungal cultivar mediating interspecies and interkingdom interactions has remained fragmented. Our omics-driven chemical, genomic, and phylogenetic study provides new insights into the volatilome and biosynthetic capabilities of the evolutionarily conserved fungal genus *Termitomyces*, which allows matching metabolites to genes and enzymes and, thus, opens a new source of unique and rare enzymatic transformations.

## INTRODUCTION

**FIG 1:**
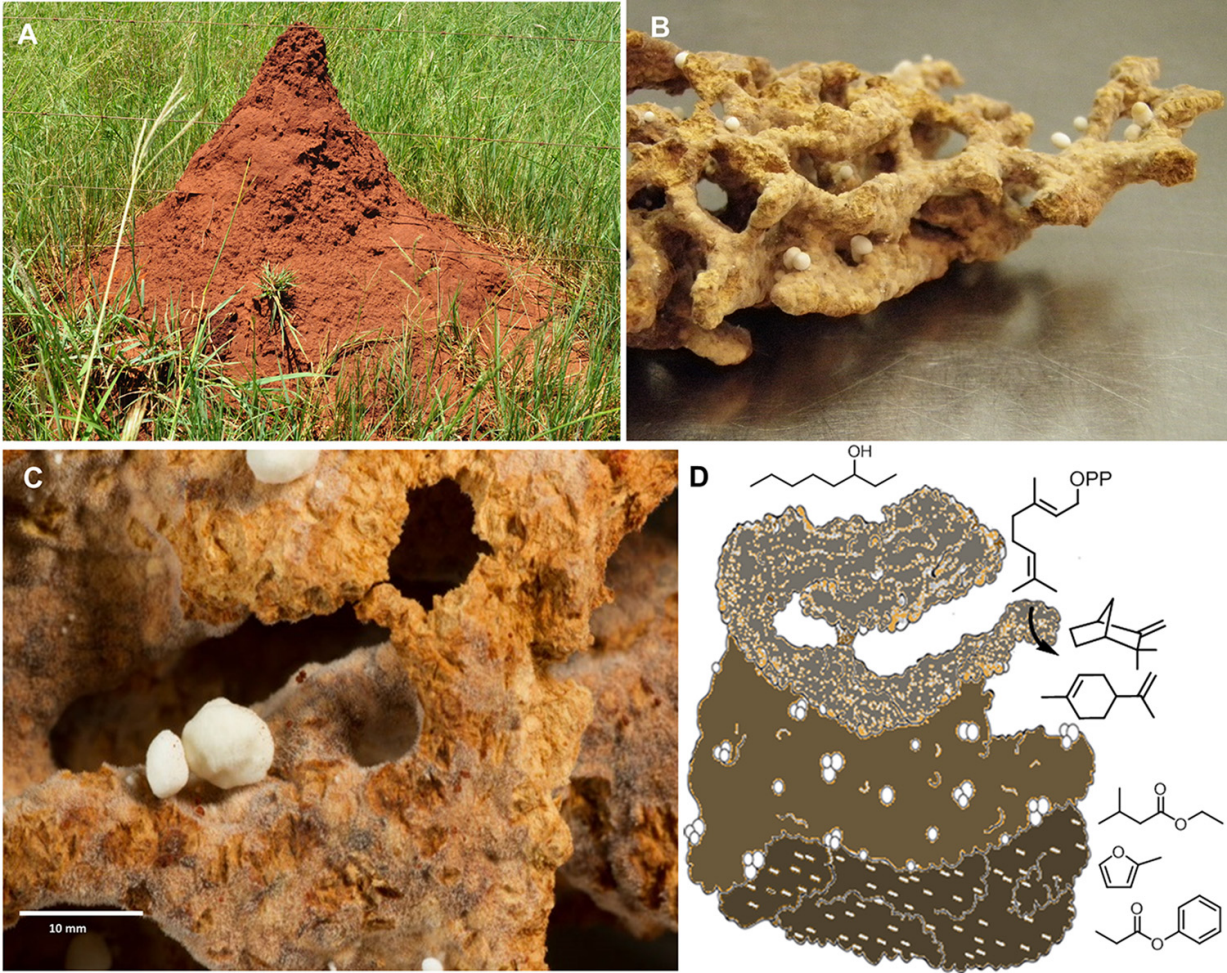
(A) Termite mound of a *Macrotermes natalensis* colony; (B) belowground garden chamber filled with fungus comb biomass; (C) closeup picture of fungus comb material pervaded with fungal biomass and pointy nodules (scale, 10 mm); and (D) schematic sketch of fungus comb emitting volatiles including monoterpenes.

*Macrotermes natalensis*, a fungus-growing termite species, has undergone an obligate mutualistic interaction with species of the basidiomycete genus *Termitomyces*. This fungus is maintained as a monoculture and is used as the primary food source by the termite colony (1, 2). Fungus-farming *M. natalensis* colonies have developed high levels of social complexity with polymorphism and division of labor across castes, similar to other nonfarming insect species. While old workers leave the nest to forage for plant material in the surrounding environment, young workers propagate the fungal monoculture of *Termitomyces* on predigested plant material crafted into comb-like structures (comb material) belowground (Fig. 1) (3–7). *Termitomyces* species mostly grow as fungal mycelium on the predigested plant biomass (fungus comb), thereby decomposing the lignocellulose-rich biomass, and occasionally differentiate into fungal nodules (1, 7). While the ecology and biology of social termites is well described, our current understandings of the molecular mechanisms that drive and orchestrate the sophisticated organization of the agricultural insect society remain fragmented (8). Communication by volatiles represents one possible mechanism that might be used by *Termitomyces* to coordinate its obligate symbiotic relation with the termite colony and its fungal life cycle, as volatiles can quickly diffuse within the subterranean fungus chambers (5). Similar to bacteria (9), fungi are known to emit a complex mixture of volatile organic compounds (VOCs), including alcohols, hydrocarbons, aldehydes, ketones, acids, esters, and terpenes that contribute to their characteristic odor (10, 11) and can be linked to their phylogenetic placement, sexual life cycle (12, 13), and parasitic, saprophytic, or symbiotic lifestyles (14–16). Only recently, first reports on the volatilome of *Termitomyces heimii* (17) and *Termitomyces* sp. strain J132 (18, 19) hinted toward the enormous capacity of *Termitomyces* species to produce a diverse set of volatiles, including terpenes. To test the hypothesis that VOCs in general, and fungal VOCs in particular, are produced within the comb environment and could serve as communication signals within the agricultural society, we analyzed the volatilome of fungal comb material and fungal nodules of *Termitomyces* species from *M. natalensis* colonies using gas chromatography-mass spectrometry (GC-MS). To identify the biosynthetic origin of the detected VOCs, we then analyzed the genomes of *Termitomyces* strains and found that members of this genus harbor a distinct set of secondary metabolite gene clusters, including an extraordinary number of genes annotated as terpene cyclases, many of which were differentially transcribed in different biological samples. Finally, we verified the enzymatic origin for the most abundant terpenes *in vitro*, thereby linking the obtained metabolomic and transcriptomic data sets.

## RESULTS AND DISCUSSION

### Volatilome of the fungus comb.

In a first experimental setup, we collected different types of biosamples (fungus comb, fungal nodules, and termite workers, with soil and air samples serving as controls) from six different *M. natalensis* colonies (see Table S1 at https://doi.org/10.6084/m9.figshare.16702471). Volatiles were collected from preweighed biosamples (*n* = 3) using solid-phase microextraction (SPME) and then analyzed by GC-MS. Obtained data sets were dereplicated using the National Institute of Standards Mass Spectral Library (NIST 2017), and peak intensities (with a match quality of at least 90%) were quantified relative to the measured sample weight and averaged signal intensities (see Fig. S1 to S3 and Tables S2 and S3 at https://doi.org/10.6084/m9.figshare.16702471).

**FIG 2:**
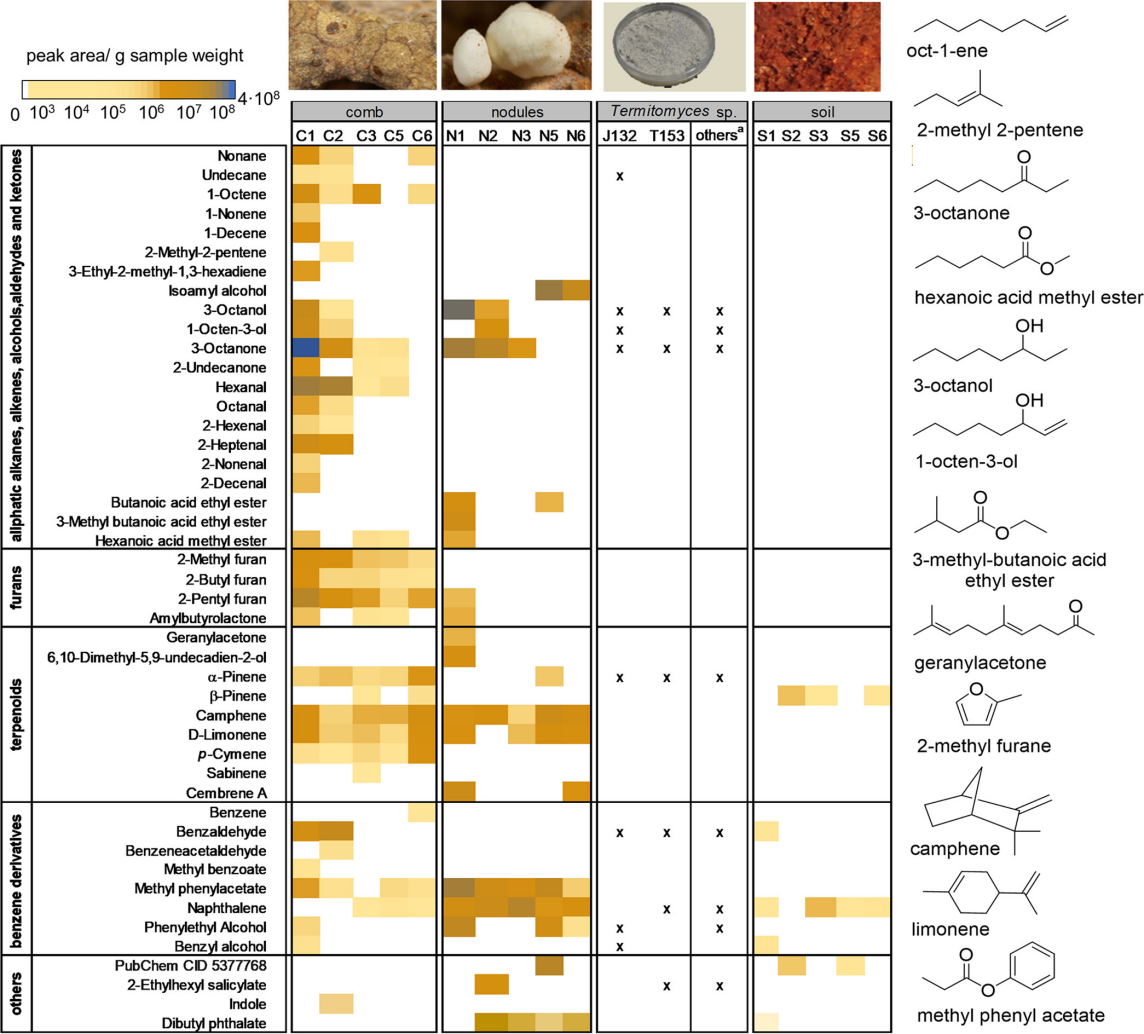
Heatmap of assigned volatiles detected from comb, nodules, and soil from termite mounds (signal intensities based on averaged peak area [*n* = 3] per g of sample) and axenic *Termitomyces* sp. strain T153 and J132 cultures (footnote a, others, combined results from literature reported volatile studies; see references 17, 18, and 23).

As depicted in Fig. 2, fungus comb samples emitted a diverse set of volatiles, including alkanes, alcohols, aliphatic aldehydes (e.g., undecane, 3-octanol, hexanal), ketones (e.g., undecanone, 3-octanone), furanes, terpenes, and aromatic metabolites. In comparison, fungal nodules emitted a less diverse volatile blend containing, e.g., isoamyl alcohol, oxidized octane derivatives (20), butanoic acid derivatives, terpenes, and, in particular, a specific set of aromatic volatiles (benzyl acetic acid methyl ester and phenylethyl alcohol), while control samples (air and soil) emitted only a few detectable volatiles, most dominantly β-pinene and naphthalene (see Tables S4 to S71 at https://doi.org/10.6084/m9.figshare.16702471).

It was particularly noteworthy that the emission of several monoterpenes was detectable from the volatile blend released by the fungus comb as well as nodules. While d-limonene and camphene were emitted from both samples, α-pinene was predominately detected from comb samples. Interestingly, β-pinene and 3-carene, common plant metabolites of pine trees, were detected in soil and air samples and might have originated from the predigested plant material of the comb or surrounding trees (21). However, most of the detected chemical features from comb and nodule samples are known fungal infochemicals emitted during fungal growth and differentiation (22), some of which were also detected in previous volatile studies of *Termitomyces* and, thus, may contribute to the characteristic scent of the fungal cultivar (18, 23). While isoamyl alcohol is known to induce morphological changes in yeast (24, 25), phenylethyl alcohol serves as an antifungal agent (26) and might have additional morphogenic functions during the fungal life cycle, as shown in the yeast Candida albicans (27). Both α-pinene and d-limonene are known microbial volatiles with antimicrobial properties (9, 16, 28) and serve as communication signals in other termite species (29, 30), while camphene is reported to have antioxidant activities (31). Only a few of the detected chemical features were deduced to be of anthropogenic or unknown origin (e.g., 2-ethylhexyl salicylate, cyclohexane, and trichloromethane) (29). To corroborate these findings and to pinpoint major termite volatiles, we also measured VOCs of 30 major workers (Tables S71 to S73). Here, several known sesquiterpenes were detectable, with β-gurjunene, gymnomitrene, and aristolene as the most abundant features, as well as smaller amounts of four terpenes, *trans*-α-bergamotene, α-pinene, α-barbatene, and β-chamigrene that were also produced by, e.g., *Termitomyces* sp. strain J132 and could be due to insects feeding on fungus biomass (see Tables S4 to S71 and Fig. S4 at https://doi.org/10.6084/m9.figshare.16702471).

### *In silico* analysis of biosynthetic gene clusters in *Termitomyces* sp.

To correlate detected volatiles to the biosynthetic capacity of *Termitomyces*, we analyzed the draft genomes of eight *Termitomyces* strains using the platform fungiSMASH (v6.0) and web service NRPSpredictor2 (see Table S73 at https://doi.org/10.6084/m9.figshare.16702471) (32, 33). Overall, each fungal genome encoded more than 20 detectable biosynthetic features related to secondary metabolism, including predominately fungal terpene cyclases (TCs), one nonreducing iterative type I polyketide synthase (NR-PKS) with conserved domain architecture (SAT-KS-AT-PT-ACP-ACP-TE), and a nonribosomal peptide synthetase (NRPS) with a conserved domain architecture (A_1_-T_1_-C_1_-T_2_-C_2_-T_3_-C_3_) and an adjacent putative cytochrome P450 monooxygenase (34). Phylogenetic analysis of the NR-PKS sequences revealed their close relationship to known orsellinic acid-producing synthases described from other basidiomycetes (35, 36), while an analysis of the detected NRPS sequences indicated their close relationship to one of the most abundant type VI basidiomycete siderophore synthetases that is assumed to produce the trimeric siderophore basidioferrin, derived from *N*^5^-acylated *N*^5^-hydroxy-l-ornithine (l-AHO) (see Table S73 and Fig. S5 and S6 at https://doi.org/10.6084/m9.figshare.16702471) (37). Thus, we deduced that both PKS and NRPS are unlikely to be accountable for any of the observed chemical features in the volatilome.

Consequently, we focused on the analysis and categorization of identified putative fungal terpene cyclase (TC) sequences based on the following general classification: (i) monoterpene cyclases (MTCs), which cyclize geranylpyrophosphate (GPP), yielding monoterpenes; (ii) sesquiterpene cyclases (STCs), which produce sesquiterpenes from farnesyl pyrophosphate (FPP); (iii) diterpene cyclases (DTCs), which use geranylgeranylpyrophosphate for product formation; and (iv) triterpene cyclases (Tri-TCs), also known as oxidosqualenecyclases (OSC)/lanosterol synthases, which catalyze cyclization of oxidosqualene into triterpenes.

**FIG 3:**
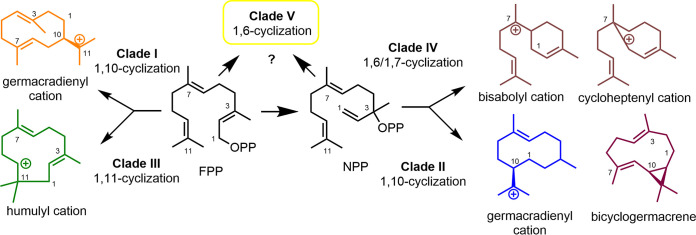
Key cyclization intermediates or products for STCs belonging to clades I to V.

Both MTCs and STCs are known to initiate terpene formation by a metal-dependent ionization and diphosphate release mechanism and are identified by the characteristic active-site motifs (DDXXD and NSE) that stabilize and guide the reaction pathway (9, 10). However, *in silico* predictions of fungal MTCs are still ambiguous due to their high similarities to fungal STC (similar active motifs, catalytic pocket, and sequence length) and the low numbers of biochemical characterized MTCs in basidiomycetes (38). In contrast, numerous fungal STCs have been characterized in basidiomycetes, and comparative studies uncovered a correlation between their phylogenetic placement and cyclization mechanism (clades I to V) (Fig. 3) (16, 39–41). In short, STCs belonging to clade I have been found to cyclize FPP via the formation of a germacradienyl cation, while STCs belonging to clade II induce a 1,10-cyclization mechanism of nerolidyl diphosphate (NPP), resulting either in bicyclogermacrene (clade IIa) or the germacradienyl cation (clade IIb), which undergoes further transformations. In contrast, enzymes belonging to clade III generally produce humulyl cation intermediates via 1,11-cyclization of FPP. Finally, enzymes of clade IV catalyze a 1,6- or 1,7-cyclization of NPP, which leads to an intermediate bisabolyl or cycloheptenyl cation, while clade V putatively includes TCs that favor only a 1,6-cyclization mechanism.

To the best of our knowledge, only 10 DTCs have been biochemically characterized so far from basidiomycetes and were found to be either mono- or bifunctional enzymes (16). While monofunctional DTCs cyclize GGPP via a type I ionization-dependent mechanism centered at the N terminus, triggering the second cyclization step by diphosphate removal (ionization-triggered reaction) (16, 39), bifunctional enzymes use both class I and class II mechanisms to form diterpenes. Here, class II activity is located at the C-terminal site and catalyzes the cyclization of GGPP by protonation of the substrate. Only recently, a third class of fungal DTC was discovered that contains proteins from the UbiA family, which can have class I diterpene cyclase activity besides prenylation activity (42, 43). Tri-TCs, which are key enzymes in ergosterol biosynthesis, carry a QW motif that determines its cyclization specificity in the transformation of squalene epoxide via a type II cyclization mechanism (9, 10). So far only four Tri-TC candidates involved in the biosynthesis of triterpenoid natural products have been characterized from different fungal species (44).

Based on these considerations, we extended our fungiSMASH survey by dedicated HMM profile searches of predicted protein sequences of TCs (45). Overall, we identified, on average, 22 distinct putative MTC/STC protein sequences per strain, which were predicted from the open reading frames in *Termitomyces* genomes, while slightly lower numbers of protein sequences for putative DTCs and Tri-TCs were identified (Table 1 and Tables S74 to S82 at https://doi.org/10.6084/m9.figshare.16702471). The number of predicted TCs varied across *Termitomyces* species, which is likely a result of various sequencing qualities and annotation approaches as well as lacking transcriptomic data, which impairs the prediction of putative proteins. Despite these ambiguities, it was important to note that genomes of free-living basidiomycetes generally encoded lower numbers of terpene cyclases, a finding suggestive of the importance of terpene-based communication mechanisms in a termite-associated lifestyle.

**TABLE 1:** Numbers of identified putative TC protein sequences in *Termitomyces* species and other model basidiomycetes

*Termitomyces* strain	Host	No. of sequences		
		M/STC^*a*^	DTC	Tri-TC
*Termitomyces*				
T153	*Macrotermes natalensis*	36	5	1
T112	*Macrotermes natalensis*	36	4	1
J132	*Macrotermes natalensis*	37	6	2
GCA_003316525	Not defined^*c*^	12	3	2
GCA_003313785	Not defined^*c*^	20	2	1
GCA_003313675	Not defined^*c*^	8	1	1
GCA_00313075	Not defined^*c*^	16	3	1
GCA_003313055	Not defined^*c*^	20	2	1
GCA_001972325	*Macrotermes gilvus*	13	1	1
Avg		22	3	1
Other^*b*^				
abp		10	3	2
lbc		10	5	3
mpi		18	13	4
scm		4	19	2

a Due to ambiguous *in silico* analyses, putative MTC and STC counts are combined.

b abp, *Agaricus bisporus* var. *burnetti*; lbc, *Laccaria bicolor*; mpi, *Moniliophthora perniciosa*; scm, *Schizophyllum commune*.

c see https://www.ncbi.nlm.nih.gov/bioproject/?term=PRJNA454572.

### Phylogenetic analysis and prediction of chemical scaffolds.

**FIG 4:**
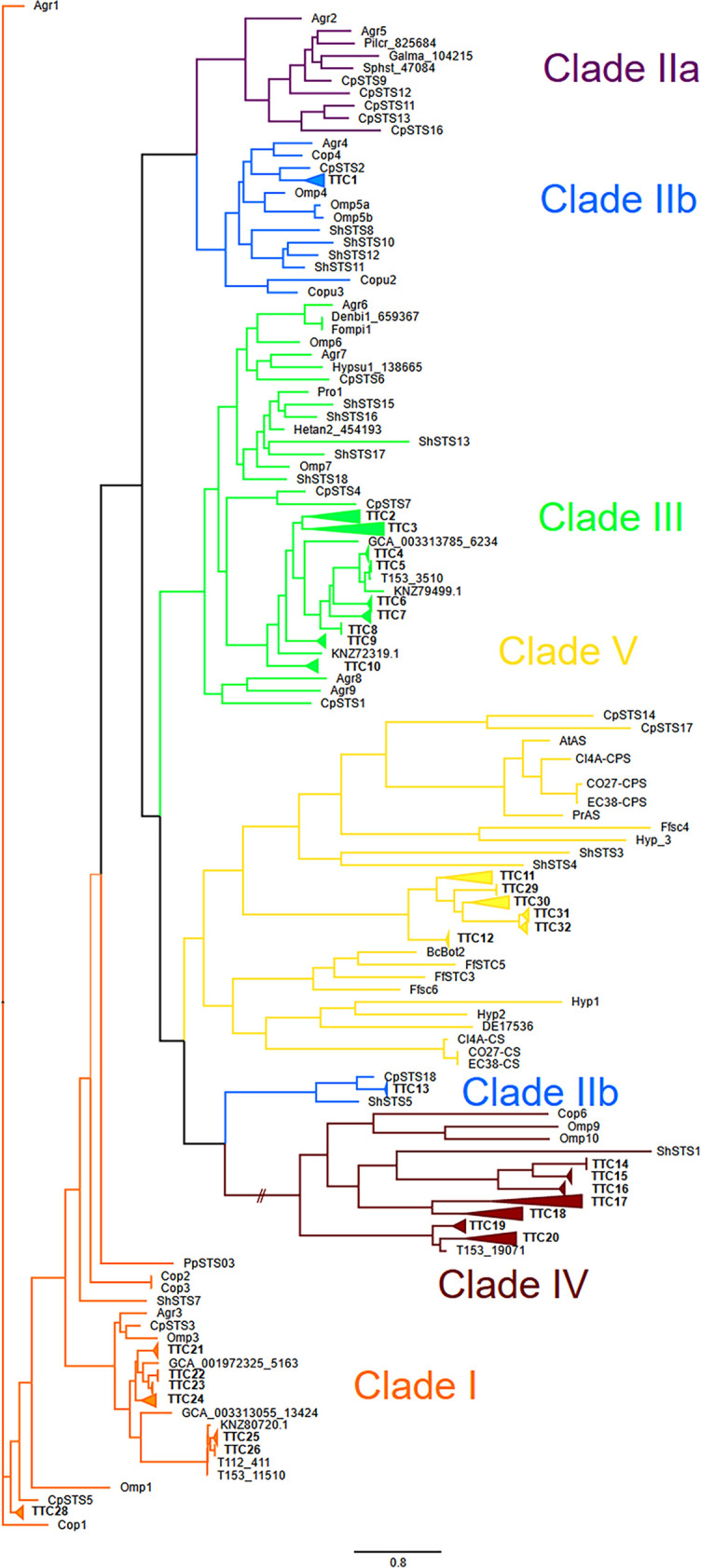
Phylogenetic tree of characterized fungal STC and putative *Termitomyces* STC protein sequences showing similarities across species (TTCs), which were grouped in clades according to their predicted cyclization mechanism (unrooted tree [based on maximum likelihood method] with branch values indicating bootstrap support of 1,000 pseudoreplicates).

To more precisely predict the putative cyclization mechanism and product scope of the identified TCs, we performed a phylogenetic analysis of all candidate sequences identified from the nine *Termitomyces* spp., as depicted in Fig. 4.

Eight terpene cyclases (TTCs; TTC21 to TTC28) showed high homologies to sequences belonging to STCs of clade I, with TTC21 to TTC26 appearing to build a *Termitomyces*-specific subclade. TTC28 from *Termitomyces* sp. strain J132 (KNZ77998; previously named STC15) (19) was biochemically characterized in previous studies and found to cyclize FPP in a 1,10-cyclization fashion via the (*E*,*E*)-germacradienyl cation to (+)-germacrene D-4-ol. We also identified two groups of TCs belonging to clade II (TTC1 and TTC13), with TTC13 being more closely related to members of clade IV, while nine TTCs (TTC2 to TTC10) were categorized as representatives of clade III and again appeared to belong to a *Termitomyces-*specific subclade. Earlier studies revealed that one representative of TTC13 (previously named STC9, from *Termitomyces* strain J132, KNZ74377) acts as a (−)-γ-cadinene synthase by cyclizing NPP at positions 1 and 10, a typical cyclization mode for clade II terpene cyclases (38). TTC13 is phylogenetically related to CpSTS18 (*Clitopilus pseudopinsitus*) and ShSTS5 (*Stereum hirsutum*), both known producers of γ-cadinene. Seven TTCs (TTC14 to TTC20) were located in clade IV, and sequence alignments of all representatives showed that the NSE motif is well conserved between Cop6, Omp9, Omp10, and all *Termitomyces* sequences, while the aspartate-rich motif (DDXXD/E; Tables S82 to S86) varied in *Termitomyces* enzymes. Based on their phylogenetic relatedness, six TTCs were assigned to clade V, forming their own subclade (TTC11, TTC12, and TTC30 to TTC32). Here, it was intriguing to note that one member of the TTC31 group (STC4; from *Termitomyces* strain J132, KNZ72568) was previously characterized to cyclize FPP via germacradienyl cation formation to the main product, (+)-intermedeol, although these enzymes were predicted to guide FPP through a 1,6-cyclization pathway (19). This example showcases again that the outlined relation between phylogenetic relatedness and cyclization mode harbors several exceptions, and the outlined predictions usually require biochemical verification (see Fig. S4 at https://doi.org/10.6084/m9.figshare.16702471).

**FIG 5:**
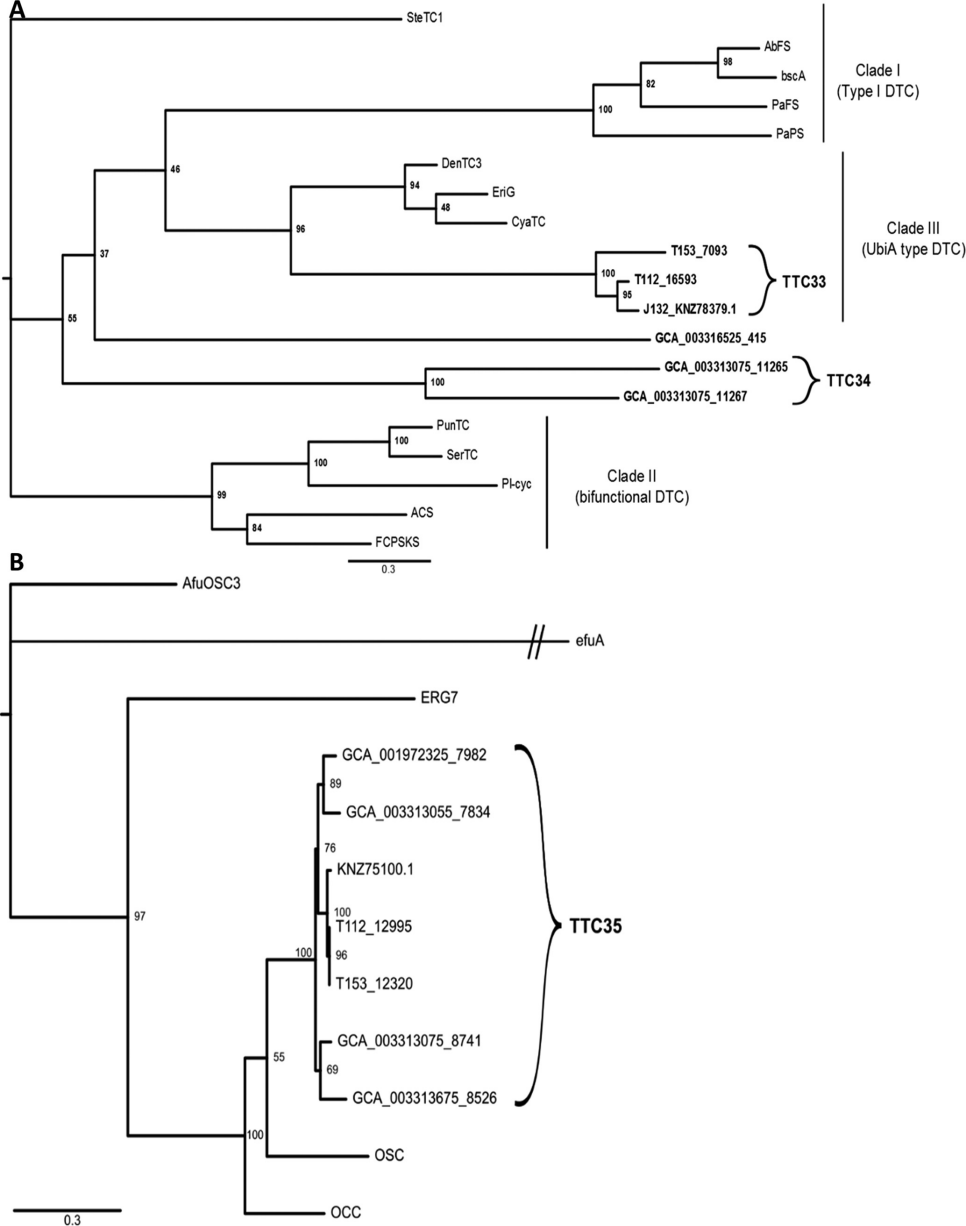
(A and B) Phylogenetic analysis of DTCs (A) and Tri-TCs (B) grouped in clades according to similarity (unrooted tree [based on maximum likelihood method] with branch values indicating bootstrap support of 1,000 pseudoreplicates).

The six identified putative DTC genes were grouped as TTC33 to TTC34 (Fig. 5A) and classified according to the classification in Li et al. (42, 43). We tentatively categorized the first group, TTC33, as UbiA-type DTCs (clade III), while TTC34 was assigned to an unclassified phylogenetic branch. Lastly, we also identified seven homologous Tri-TC candidate sequences based on our similarity searches (Fig. 5B), which were grouped as TTC35 and found to be closely related to OSC from Ganoderma lucidum and known to produce lanosterol (46), the precursor for ergosterol (47) and other triterpene-based metabolites (e.g., ganoderic acids) (10, 48).

### RNA-seq analysis.

**FIG 6:**
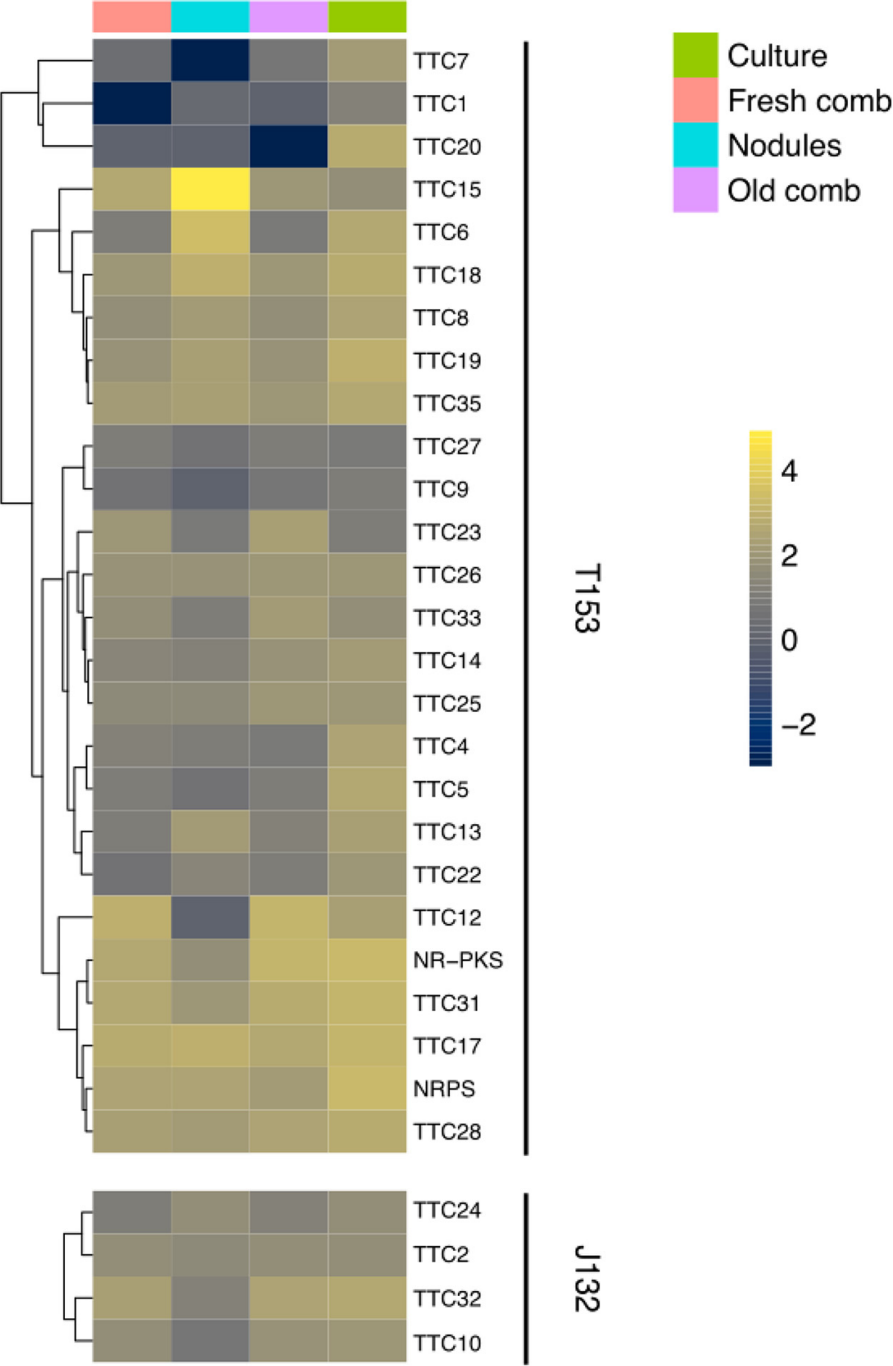
Heatmap of expression levels of putative natural product biosynthetic genes from *Termitomyces* species from *Macrotermes* colony Mn156 (49) under natural conditions (nodules, fresh, and old comb) and plate culture of *Termitomyces* sp. strains J132 and T153 (green). Transcript abundances are depicted as log_10_ gene expression values, and color schemes were generated by viridis (version 0.5.1).

Subsequently, we analyzed the relative expression levels of natural product biosynthetic-related gene sequences in transcriptome sequencing (RNA-seq) data obtained from cultured *Termitomyces* sp. strains 153 and J132 as well as fresh and old fungus comb and nodules on which young workers feed (Fig. 6 and Fig. S7 and S8 at https://doi.org/10.6084/m9.figshare.16702471) (49). Intriguingly, a differential expression pattern of TTCs was observed, with some candidate sequences mostly expressed in comb and lab cultures but not in nodules, while others were expressed in all samples. Intriguingly, one candidate sequence (TTC15) was predominately expressed in fungal nodules, while only moderate to low expression levels were detectable within the comb and agar cultures. In contrast, NRPS- and NR-PKS-related gene sequences were expressed with similar abundance under all conditions and confirmed by RT-PCR of a fungal monoculture (Fig. S9 at https://doi.org/10.6084/m9.figshare.16702471).

### Biochemical analysis of TTC15-T153.

**FIG 7:**
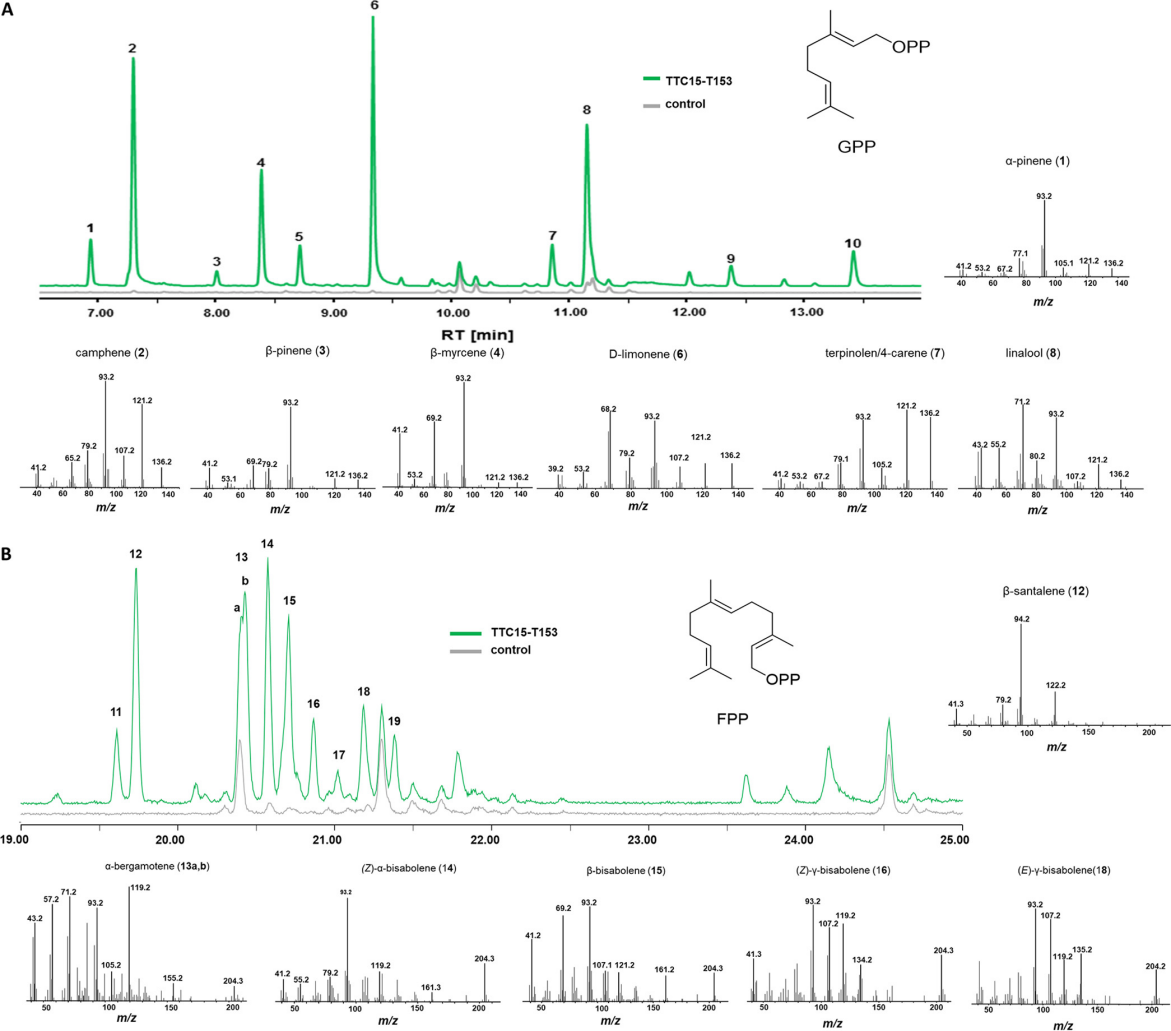
GC chromatogram of extracts obtained from purified enzyme TTC15-T153 and control sample incubated with GPP (A) and FPP (B) and selected MS spectra of tentatively identified compounds (*y* axis, relative abundance).

We then hypothesized that some of the terpenes detected in the headspace of comb and nodules (e.g., limonene/camphene, Fig. 2) were produced from proteins encoded by the highly expressed TTC15 sequence (Fig. 6). To test our hypothesis, we heterologously expressed a homologous gene sequence of TTC15 identified in *Termitomyces* sp. strain T153 (TTC15-T153). The coding sequence was synthesized from mRNA predictions, cloned in a pET28a vector, and heterologously expressed in Escherichia coli BL21 cells. The hexahistidine fusion protein of enzyme TTC15-T153 (36.5 kDa; native protein, 34.3 kDa) was purified on a Ni^2+^-NTA-affinity column with a yield of 14 mg protein per 100 ml expression culture (Fig. S10 at https://doi.org/10.6084/m9.figshare.16702471). The purified protein was incubated at 30°C for 2 h with GPP, FPP, or GGPP in the presence of Mg^2+^ as a cofactor. Following the reaction, the produced compounds were extracted with hexane and analyzed by GC-MS, and detected products were tentatively assigned using the NIST 2017 database. While incubation with GGPP resulted in no detectable product formation, transformation of GPP by TTC15-T153 resulted in the formation of several major monoterpenes, such as camphene (2), β-myrcene (4), *d-limonene* (6), and linalool (8) (Fig. 7A and Table S6 and Fig. S10). Additionally, α-pinene (1), β-pinene (3), α-phellandrene (5), terpinolene/4-carene (7), 3-methyl camphenilanol (9), and α-terpineol (10) were formed as minor products (see Fig. S11 at https://doi.org/10.6084/m9.figshare.16702471).

In contrast, incubation of TTC15-T153 with FPP yielded eight major terpenes, including β-/epi-β-santalene (12), α-bergamotenes (13a/b), and a mixture of bisabolene isomers (14, 15, 16, 18, and 19) as well as two minor products [(*E*)-β-farnesene (11) and β-sesquiphellandrene (17)] (Fig. 7B and Fig. S11 and Tables S87 and S88 at https://doi.org/10.6084/m9.figshare.16702471). The observed bifunctional activity of TTC15-T153 was intriguing, as only one other enzyme from basidiomycetes (Agr4 from *Agrocybe aegerita*) has been characterized to convert GPP as well as FPP into cyclic terpenes (40).

**FIG 8:**
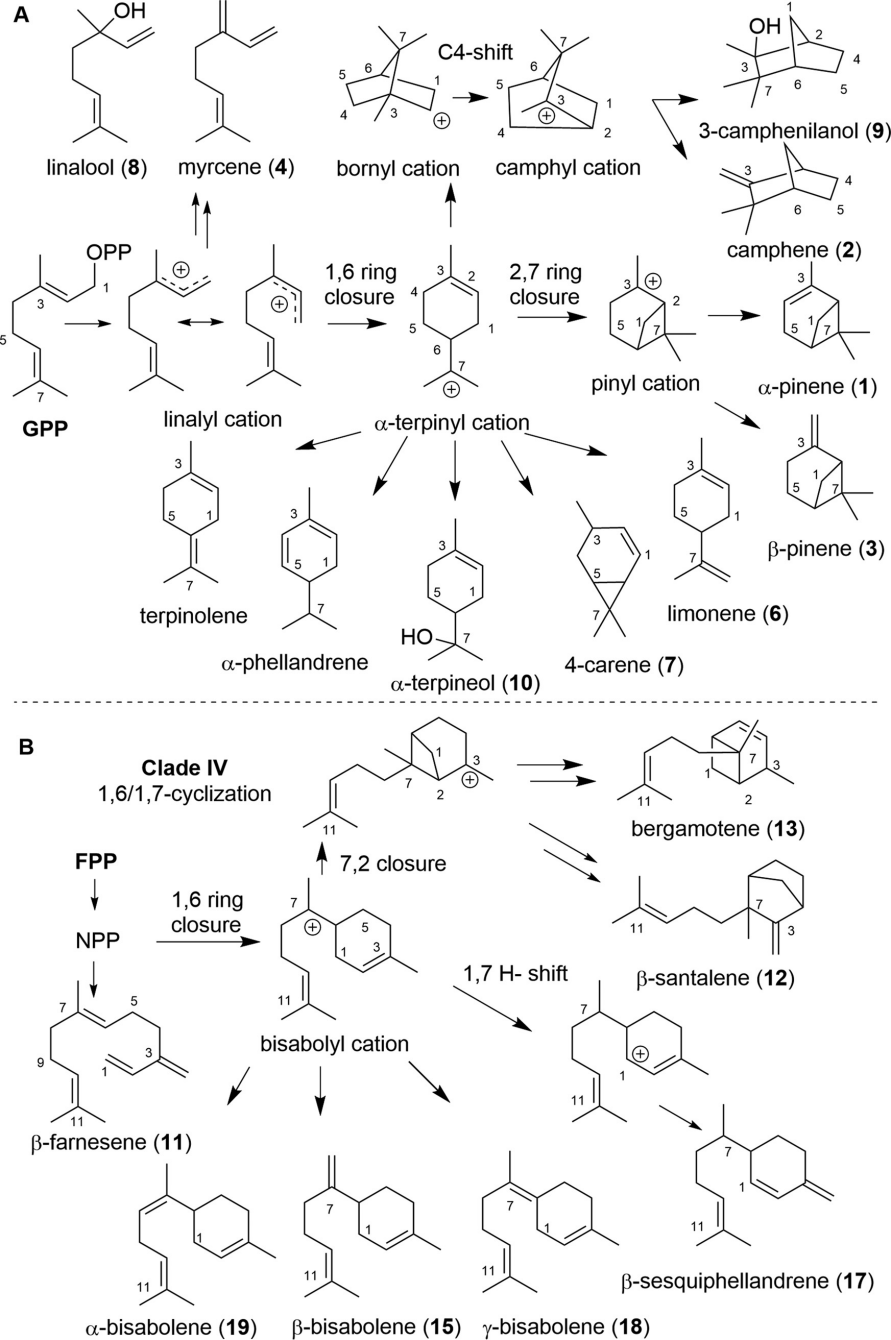
Putative enzymatic conversion of GPP (A) and FPP (B) by terpene cyclase TTC15-T153.

The observed product spectrum of GPP conversion by TTC15-T153 can be explained by formation of the linalyl cation (Fig. 8A), which either serves as a precursor for the linear monoterpenes linalool (8) and myrcene (4) or is further converted into the α-terpinyl cation, the intermediate precursor of terpinolene, α-phellandrene, α-terpineol (10), 4-carene (7), and limonene (6) (50, 51). Once the α-terpinyl cation is formed, it could also be further enzymatically converted via a 3,7-ring closure to the bornyl cation, which, after Wagner-Meerwein rearrangement, deprotonation, or addition of water, yields camphene (2) or 3-camphenilanol (9) (52). Furthermore, enzymatic transformation of α-terpinyl cation to the pinyl cation results in the formation of the observed α- and β-pinene (1 and 3) (53).

In the presence of FPP, TTC15-T153 likely converts FPP via NPP into a bisabolyl cation (Fig. 8B), which then results in the formation of a β-sesquiphellandrene and bisabolene blend (54). Starting from the bisabolyl cation, a 7,2-ring closure and deprotonation event can also lead to the formation of (*E*)-α-bergamotene, while β-santalene is formed after a sequence of rearrangements and a final deprotonation step (54–57).

Comparative analysis of TTC15-T153 and related sequences from clade TTC15 enzymes revealed that the first metal binding motif differs from the common DDXXD/E amino acid composition, which might cause the acceptance of both GPP and FPP as substrates by TTC15-T153, causing the formation of the different terpene blends.

### Conclusions.

In this study, we elaborated on the hypothesis that the fungal cultivar of *Macrotermitinae* termites emits specific volatiles (odor) that allows *Termitomyces* to orchestrate its complex life cycle within the fungal garden and its symbiotic relation with the termite host. To test the hypothesis, we combined volatile studies of fungal nodules and comb, in which the fungal cultivar *Termitomyces* resides, with phylogenetic analyses, gene expression, and molecular biological studies.

Our finding that *Termitomyces* thriving in fungus comb material emits a specific volatilome with α-pinene, camphene, and d-limonene among the most abundant terpenes (5–10) correlated with the identification of an above average repertoire of different types of terpene cyclases with conserved candidate sequences across *Termitomyces* species. While TCs from different *Termitomyces* strains showed high homologies to each other, they grouped into distinct phylogenetic branches compared to other TCs reported from other fungal genera. The detailed comparative analysis also uncovered that phylogenetic relatedness was not always correlated with the predicted cyclization mode, and more detailed biochemical studies on fungal TCs are required to solidify the correlations or to uncover the characteristics of their deviations. Based on RNA-seq data analysis, we verified that a congener of the highly expressed enzyme TTC15-T153 accepts GPP as well as FPP as substrates, causing the formation of more than 20 different terpenes, most of which were also detected in the volatile blends of collected biosamples and may contribute significantly to the characteristic odor of *Termitomyces*. From a chemical-ecological perspective, these findings were highly intriguing, as the two major GPP-derived products of TTC15-T153, d-limonene and camphene, were identified within the fungus comb volatilome and are known to be metabolites exchanged within symbiotic systems (14, 58). This study represents, to the best of our knowledge, the second characterized example of a bifunctional mono-/sesquiterpene cyclase in basidiomycetes. The high expression level of the coding sequence TTC15 in comb and nodules as well as natural abundance of these characteristic monoterpenes points toward a central role in termite symbiosis. Overall, our combined studies allowed us, for the first time, to link and verify produced metabolites within the complex community with their genetic basis, transcriptional pattern, and conserved biosynthetic origin and improve our current understanding of the chemical language orchestrating this complex farming symbiosis.

## MATERIALS AND METHODS

### Volatile analysis.

For volatile sampling, different parts of *M. natalensis* nests from different areas of Pretoria (South Africa) were collected in preweighed amber glass vials directly in the field. Soil, air, and fungus comb samples were collected in 40-ml amber glass vials, and nodules were separated from the fungus comb using clean forceps and collected in 1-ml amber glass vials, both equipped with a cap containing a silicone septum. The vials were closed tightly and kept at 4°C until volatile extraction. Each sample was collected in triplicates, if not stated differently. Headspace sampling was performed using solid-phase microextraction (SPME). After penetrating the silicone septum of the vial cap using an SPME fiber holder, a conditioned SPME fiber coated with 100 μm polymethylsiloxane (Supelco) was exposed to the headspace in the vial for 20 min at room temperature. Room temperature and sample weight were recorded each time before measuring. The SPME fiber was then directly injected into the inlet of an Agilent 7890B gas chromatograph coupled to a 7977MSD quadrupole mass spectrometer. Thermal desorption was achieved at 250°C. Compound separation was achieved using a DB wax GC column (length, 20 m; inner diameter, 0.18 mm; and film thickness, 0.18 μm [J&W]). Separation was achieved with a 5-min hold at 50°C, followed by a linear temperature increase of 10°C/min to 250°C. The column was reconditioned with a 2-min hold at 250°C. Data analysis and peak integration were performed using the program MSD ChemStation (version F.01.03.2357). Metabolites were tentatively identified based on comparison of mass spectra and retention times to the National Institute of Standards mass spectral library (NIST 2017). After peak detection and integration, metabolites with a database match quality of at least 90% were taken for further analysis. Thereafter, the peak area was normalized to the sample weight for each metabolite (area per gram) and integration values used to generate a heat map of averaged values (see the supplemental material at https://doi.org/10.6084/m9.figshare.16702471).

### Identification of putative NRPS and PKS and phylogenetic analysis.

Identified enzymes were aligned to characterized fungal sequences from the same class in MEGAX by MUSCLE algorithm. Phylogenetic trees were generated with IQ TREE, calculating 1,000 bootstrap replicates (see the supplemental material at https://doi.org/10.6084/m9.figshare.16702471).

### Identification of putative terpene cyclases and phylogenetic analysis.

Putative terpene cyclases in *Termitomyces* species were identified by HMMsearch of HMMprofiles from characterized fungal terpene cyclases against predicted proteins from different *Termitomyces* species. Identified terpene cyclases that matched specific criteria for each class of terpene cyclases were aligned to characterized fungal sequences from the same class in MEGAX by MUSCLE algorithm. Phylogenetic trees were generated with IQ TREE, calculating 1,000 bootstrap replicates. Bioinformatic analyses were performed on the Galaxy EU webserver (59) (see the supplemental material at https://doi.org/10.6084/m9.figshare.16702471).

### RNA-seq data acquisition and processing.

*Termitomyces* RNA-seq data for fresh comb (SRR5944783), old comb (SRR5944781), and nodules (SRR5944782) from *Macrotermes* colony Mn156 were downloaded from the European Nucleotide Archive, and RNA-seq data were from cultured *Termitomyces* sp. strain T153 (PRJNA529327) and J132 (PRJNA193471) (18). Identified terpene cyclase sequences from *Termitomyces* genomes T153 and J132 were prepared as separate references for Bowtie version 1.2.2 14 using rsem-prepare-reference in RSEM version 1.3.1.15. RNA-seq reads were then aligned to each reference genome individually, and expression levels were calculated using rsem-calculate-expression in RSEM version 1.2.2.15 (see the supplemental material at https://doi.org/10.6084/m9.figshare.16702471).

### Expression analysis.

Expression levels calculated by RSEM were imported separately for each reference genome into R version 3.6.3 (60) using tximport (version 1.12.3) (61) and DESeq2 (version 1.24.0) (62). Genes with <10 transcripts were then excluded from the data set. The remaining expression data were then plot using a log_10_ transformation, and Z scores were calculated for each row individually and then plot using pheatmap version 1.0.12 (63) with color-blind-accessible color scales from viridis version 0.6.1 (64) in R version 3.6.3 (see the supplemental material at https://doi.org/10.6084/m9.figshare.16702471).

### Heterologous expression.

The gene sequence of TTC15-153 was codon optimized for E. coli and synthesized in a pET28a(+) vector (BioCat). After transformation of E. coli BL21 cells with the plasmid pET28-TTC15, the protein was heterologously produced and purified by immobilized metal affinity chromatography for investigation of the enzyme function. This yielded the successful isolation of a 36.5-kDa protein. For assaying, the enzyme was incubated with GPP, (*E*,*E*)-FPP, and GGPP as a substrate and 10 mM Mg^2+^ as the cofactor. Hexane extracts from the reaction were directly investigated by GC-MS analysis, and compounds were identified from the NIST 2017 database in the MSD ChemStation (version F.01.03.2357) software (see the supplemental material at https://doi.org/10.6084/m9.figshare.16702471).

## References

[1] Wisselink M, Aanen DK, van’t Padje A. 2020. The longevity of colonies of fungus-growing termites and the stability of the symbiosis. Insects 11:527. doi:10.3390/insects11080527. PMC7469218 32823564

[2] Poulsen M, Hu H, Li C, Chen Z, Xu L, Otani S, Nygaard S, Nobre T, Klaubauf S, Schindler PM, Hauser F, Pan H, Yang Z, Sonnenberg ASM, de Beer ZW, Zhang Y, Wingfield MJ, Grimmelikhuijzen CJP, de Vries RP, Korb J, Aanen DK, Wang J, Boomsma JJ, Zhang G. 2014. Complementary symbiont contributions to plant decomposition in a fungus-farming termite. Proc Natl Acad Sci USA 111:14500–14505. doi:10.1073/pnas.1319718111. 25246537 PMC4209977

[3] Mitaka Y, Mori N, Matsuura K. 2017. Multi-functional roles of a soldier-specific volatile as a worker arrestant primer pheromone and an antimicrobial agent in a termite. Proc R Soc B doi:10.1098/rspb.2017.1134. PMC5543234 28747483

[4] Gershenzon J, Dudareva N. 2007. The function of terpene natural products in the natural world. Nat Chem Biol 3:408–414. doi:10.1038/nchembio.2007.5. 17576428

[5] Werner S, Polle A, Brinkmann N. 2016. Belowground communication: impacts of volatile organic compounds (VOCs) from soil fungi on other soil-inhabiting organisms. Appl Microbiol Biotechnol 100:8651–8665. doi:10.1007/s00253-016-7792-1. 27638017

[6] Tumlinson JH. 2014. The importance of volatile organic compounds in ecosystem functioning. J Chem Ecol 40:212–213. doi:10.1007/s10886-014-0399-z. 24619729

[7] Li H, Young SE, Poulsen M, Currie CR. 2021. Symbiont-mediated digestion of plant biomass in fungus-farming insects. Annu Rev Entomol 66:297–316. doi:10.1146/annurev-ento-040920-061140. 32926791

[8] Schmidt S, Kildgaard S, Guo H, Beemelmanns C, Poulsen M. 2021. The chemical ecology of the fungus-farming termite symbiosis. Nat Prod Rep doi:10.1039/D1NP00022E. PMC8865390 34879123

[9] Rudolf JD, Alsup TA, Xu B, Li Z. 2021. Bacterial terpenome. Nat Prod Rep 38:905–980. doi:10.1039/d0np00066c. 33169126 PMC8107197

[10] Quin MB, Flynn CM, Schmidt-Dannert C. 2014. Traversing the fungal terpenome. Nat Prod Rep 31:1449–1473. doi:10.1039/c4np00075g. 25171145 PMC4167380

[11] Kramer R, Abraham W-R. 2012. Volatile sesquiterpenes from fungi: what are they good for? Phytochem Rev 11:15–37. doi:10.1007/s11101-011-9216-2.

[12] Freihorst D, Brunsch M, Wirth S, Krause K, Kniemeyer O, Linde J, Kunert M, Boland W, Kothe E. 2018. Smelling the difference: transcriptome, proteome and volatilome changes after mating. Fungal Genet Biol 112:2–11. doi:10.1016/j.fgb.2016.08.007. 27593501

[13] Nemcovic M, Jakubíková L, Víden I, Farkas V. 2008. Induction of conidiation by endogenous volatile compounds in *Trichoderma* spp. FEMS Microbiol Lett 284:231–236. doi:10.1111/j.1574-6968.2008.01202.x. 18510561

[14] Hung R, Lee S, Bennett JW. 2015. Fungal volatile organic compounds and their role in ecosystems. Appl Microbiol Biotechnol 99:3395–3405. doi:10.1007/s00253-015-6494-4. 25773975

[15] Guo Y, Jud W, Weikl F, Ghirardo A, Junker RR, Polle A, Benz JP, Pritsch K, Schnitzler J-P, Rosenkranz M. 2021. Volatile organic compound patterns predict fungal trophic mode and lifestyle. Commun Biol 4:673. doi:10.1038/s42003-021-02198-8. 34083721 PMC8175423

[16] Dickschat JS. 2017. Fungal volatiles—a survey from edible mushrooms to moulds. Nat Prod Rep 34:310–328. doi:10.1039/c7np00003k. 28205661

[17] Yang G, Ahmad F, Liang S, Fouad H, Guo M, Gaal HA, Mo J. 2020. *Termitomyces heimii* associated with fungus-growing termite produces volatile organic compounds (VOCs) and lignocellulose-degrading enzymes. Appl Biochem Biotechnol 192:1270–1283. doi:10.1007/s12010-020-03376-w. 32720080

[18] Schalk F, Gostinčar C, Kreuzenbeck NB, Conlon BH, Sommerwerk E, Rabe P, Burkhardt I, Krüger T, Kniemeyer O, Brakhage AA, Gunde-Cimerman N, de Beer ZW, Dickschat JS, Poulsen M, Beemelmanns C. 2021. The termite fungal cultivar *Termitomyces* combines diverse enzymes and oxidative reactions for plant biomass conversion. mBio 12:e03551-20. doi:10.1128/mBio.03551-20. PMC8262964 34126770

[19] Burkhardt I, Kreuzenbeck NB, Beemelmanns C, Dickschat JS. 2019. Mechanistic characterization of three sesquiterpene synthases from the termite-associated fungus *Termitomyces*. Org Biomol Chem 17:3348–3355. doi:10.1039/c8ob02744g. 30693926

[20] Okull DO, Beelman RB, Gourama H. 2003. Antifungal activity of 10-oxo-trans-8-decenoic acid and 1-octen-3-ol against *Penicillium expansum* in potato dextrose agar medium. J Food Prot 66:1503–1505. doi:10.4315/0362-028x-66.8.1503. 12929847

[21] Mazid M, Khan TA, Mohammad F. 2011. Role of secondary metabolites in defense mechanisms of plants. Biol Med 3:232–249.

[22] Xia Y, Zhang B, Li W, Xu G. 2011. Changes in volatile compound composition of *Antrodia camphorata* during solid state fermentation. J Sci Food Agric 91:2463–2470. doi:10.1002/jsfa.4488. 21823126

[23] Katariya L, Ramesh PB, Gopalappa T, Desireddy S, Bessière JM, Borges RM. 2017. Fungus-farming termites selectively bury weedy fungi that smell different from crop fungi. J Chem Ecol 43:986–995. doi:10.1007/s10886-017-0902-4. 29124530

[24] Kern K, Nunn CD, Pichová A, Dickinson JR. 2004. Isoamyl alcohol-induced morphological change in *Saccharomyces cerevisiae* involves increases in mitochondria and cell wall chitin content. FEMS Yeast Res 5:43–49. doi:10.1016/j.femsyr.2004.06.011. 15381121

[25] Martins M, Henriques M, Azeredo J, Rocha SM, Coimbra MA, Oliveira R. 2007. Morphogenesis control in *Candida albicans* and *Candida dubliniensis* through signaling molecules produced by planktonic and biofilm cells. Eukaryot Cell 6:2429–2436. doi:10.1128/EC.00252-07. 17981993 PMC2168255

[26] Liu P, Cheng Y, Yang M, Liu Y, Chen K, Long C, Deng X. 2014. Mechanisms of action for 2-phenylethanol isolated from Kloeckera apiculata in control of *Penicillium* molds of citrus fruits. BMC Microbiol 14:242. doi:10.1186/s12866-014-0242-2. 25230758 PMC4177429

[27] Chauhan NM, Raut JS, Karuppayil SM. 2011. A morphogenetic regulatory role for ethyl alcohol in *Candida albicans*. Mycoses 54:e697–e703. doi:10.1111/j.1439-0507.2010.02002.x. 21605190

[28] Thakre A, Zore G, Kodgire S, Kazi R, Mulange S, Patil R, Shelar A, Santhakumari B, Kulkarni M, Kharat K, Karuppayil SM. 2018. Limonene inhibits *Candida albicans* growth by inducing apoptosis. Med Mycol 56:565–578. doi:10.1093/mmy/myx074. 29420815

[29] Sobotník J, Jirosová A, Hanus R. 2010. Chemical warfare in termites. J Insect Physiol 56:1012–1021. doi:10.1016/j.jinsphys.2010.02.012. 20223240

[30] Valterová I, Vrkoč J, Norin T. 1993. The enantiomeric composition of monoterpene hydrocarbons in the defensive secretions of *Nasutitermes termites* (Isoptera): inter- and intraspecific variations. Chemoecology 4:120–123. doi:10.1007/BF01241682.

[31] Hachlafi NE, Aanniz T, Menyiy NE, Baaboua AE, El Omari N, Balahbib A, Shariati MA, Zengin G, Fikri-Benbrahim K, Bouyahya A. 2021. *In vitro* and *in vivo* biological investigations of camphene and its mechanism insights: a review. Food Rev Int doi:10.1080/87559129.2021.1936007.

[32] Röttig M, Medema MH, Blin K, Weber T, Rausch C, Kohlbacher O. 2011. NRPSpredictor2–a web server for predicting NRPS adenylation domain specificity. Nucleic Acids Res 39:W362–W367. doi:10.1093/nar/gkr323. 21558170 PMC3125756

[33] Blin K, Shaw S, Kloosterman AM, Charlop-Powers Z, van Wezel GP, Medema MH, Weber T. 2021. antiSMASH 6.0: improving cluster detection and comparison capabilities. Nucleic Acids Res 49:W29–W35. doi:10.1093/nar/gkab335. 33978755 PMC8262755

[34] Gressler M, Löhr NA, Schäfer T, Lawrinowitz S, Seibold PS, Hoffmeister D. 2021. Mind the mushroom: natural product biosynthetic genes and enzymes of Basidiomycota. Nat Prod Rep 38:702–722. doi:10.1039/d0np00077a. 33404035

[35] Lackner G, Bohnert M, Wick J, Hoffmeister D. 2013. Assembly of melleolide antibiotics involves a polyketide synthase with cross-coupling activity. Chem Biol 20:1101–1106. doi:10.1016/j.chembiol.2013.07.009. 23993460

[36] Braesel J, Fricke J, Schwenk D, Hoffmeister D. 2017. Biochemical and genetic basis of orsellinic acid biosynthesis and prenylation in a stereaceous basidiomycete. Fungal Genet Biol 98:12–19. doi:10.1016/j.fgb.2016.11.007. 27903443

[37] Brandenburger E, Gressler M, Leonhardt R, Lackner G, Habel A, Hertweck C, Brock M, Hoffmeister D, Elliot MA. 2017. A highly conserved basidiomycete peptide synthetase produces a trimeric hydroxamate siderophore. Appl Environ Microbiol 83:e01478-17. doi:10.1128/AEM.01478-17. 28842536 PMC5648918

[38] Shaw JJ, Berbasova T, Sasaki T, Jefferson-George K, Spakowicz DJ, Dunican BF, Portero CE, Narváez-Trujillo A, Strobel SA. 2015. Identification of a fungal 1,8-cineole synthase from *Hypoxylon* sp. with specificity determinants in common with the plant synthases. J Biol Chem 290:8511–8526. doi:10.1074/jbc.M114.636159. 25648891 PMC4375501

[39] Jia Q, Chen X, Köllner TG, Rinkel J, Fu J, Labbé J, Xiong W, Dickschat JS, Gershenzon J, Chen F. 2019. Terpene synthase genes originated from bacteria through horizontal gene transfer contribute to terpenoid diversity in fungi. Sci Rep 9:9223. doi:10.1038/s41598-019-45532-1. 31239482 PMC6592883

[40] Zhang C, Chen X, Orban A, Shukal S, Birk F, Too H-P, Rühl M. 2020. *Agrocybe aegerita* serves as a gateway for identifying sesquiterpene biosynthetic enzymes in higher fungi. ACS Chem Biol 15:1268–1277. doi:10.1021/acschembio.0c00155. 32233445

[41] Nagamine S, Liu C, Nishishita J, Kozaki T, Sogahata K, Sato Y, Minami A, Ozaki T, Schmidt-Dannert C, Maruyama JI, Oikawa H. 2019. Ascomycete *Aspergillus oryzae* is an efficient expression host for production of basidiomycete terpenes by using genomic DNA sequences. Appl Environ Microbiol 85:e00409-19. doi:10.1128/AEM.00409-19. 31101615 PMC6643257

[42] Yang Y-l, Zhang S, Ma K, Xu Y, Tao Q, Chen Y, Chen J, Guo S, Ren J, Wang W, Tao Y, Yin W-B, Liu H. 2017. Discovery and characterization of a new family of diterpene cyclases in bacteria and fungi. Angew Chem Int Ed Engl 56:4749–4752. doi:10.1002/anie.201700565. 28371074

[43] Li X-L, Xu Y-X, Li Y, Zhang R, Hu T-Y, Su P, Zhou M, Tang T, Zeng Y, Yang Y-L, Gao W. 2019. Rapid discovery and functional characterization of diterpene synthases from basidiomycete fungi by genome mining. Fungal Genet Biol 128:36–42. doi:10.1016/j.fgb.2019.03.007. 30905831

[44] Kuhnert E, Li Y, Lan N, Yue Q, Chen L, Cox RJ, An Z, Yokoyama K, Bills GF. 2018. Enfumafungin synthase represents a novel lineage of fungal triterpene cyclases. Environ Microbiol 20:3325–3342. doi:10.1111/1462-2920.14333. 30051576 PMC6237087

[45] Quin MB, Flynn CM, Wawrzyn GT, Choudhary S, Schmidt-Dannert C. 2013. Mushroom hunting by using bioinformatics: application of a predictive framework facilitates the selective identification of sesquiterpene synthases in Basidiomycota. Chembiochem 14:2480–2491. doi:10.1002/cbic.201300349. 24166732 PMC3866635

[46] Shang CH, Shi L, Ren A, Qin L, Zhao MW. 2010. Molecular cloning characterization and differential expression of a lanosterol synthase gene from Ganoderma lucidum. Biosci Biotechnol Biochem 74:974–978. doi:10.1271/bbb.90833. 20460708

[47] Dupont S, Lemetais G, Ferreira T, Cayot P, Gervais P, Beney L. 2012. Ergosterol biosynthesis: a fungal pathway for life and land? Evolution 66:2961–2968. doi:10.1111/j.1558-5646.2012.01667.x. 22946816

[48] Hu Y, Ahmed S, Li J, Luo B, Gao Z, Zhang Q, Li X, Hu X. 2017. Improved ganoderic acids production in *Ganoderma lucidum* by wood decaying components. Sci Rep 7:46623. doi:10.1038/srep46623. 28422185 PMC5395960

[49] da Costa RR, Hu H, Pilgaard B, Vreeburg SME, Schückel J, Pedersen KSK, Kračun SK, Busk PK, Harholt J, Sapountzis P, Lange L, Aanen DK, Poulsen M. 2018. Enzyme activities at different stages of plant biomass decomposition in three species of fungus-growing termites. Appl Environ Microbiol 84:e01815-17. doi:10.1128/AEM.01815-17. 29269491 PMC5812949

[50] Degenhardt J, Köllner TG, Gershenzon J. 2009. Monoterpene and sesquiterpene synthases and the origin of terpene skeletal diversity in plants. Phytochemistry 70:1621–1637. doi:10.1016/j.phytochem.2009.07.030. 19793600

[51] Schilmiller AL, Schauvinhold I, Larson M, Xu R, Charbonneau AL, Schmidt A, Wilkerson C, Last RL, Pichersky E. 2009. Monoterpenes in the glandular trichomes of tomato are synthesized from a neryl diphosphate precursor rather than geranyl diphosphate. Proc Natl Acad Sci USA 106:10865–10870. doi:10.1073/pnas.0904113106. 19487664 PMC2705607

[52] Croteau R, Gershenzon J, Wheeler CJ, Satterwhite DM. 1990. Biosynthesis of monoterpenes: stereochemistry of the coupled isomerization and cyclization of geranyl pyrophosphate to camphane and isocamphane monoterpenes. Arch Biochem Biophys 277:374–381. doi:10.1016/0003-9861(90)90593-n. 2178556

[53] Hyatt DC, Croteau R. 2005. Mutational analysis of a monoterpene synthase reaction: altered catalysis through directed mutagenesis of (−)-pinene synthase from *Abies grandis*. Arch Biochem Biophys 439:222–233. doi:10.1016/j.abb.2005.05.017. 15978541

[54] Rinkel J, Dickschat JS. 2019. Stereochemical investigations on the biosynthesis of achiral (Z)y-bisabolene in *Cryptosporangium arvum*. Beilstein J Org Chem 15:789–794. doi:10.3762/bjoc.15.75. 30992727 PMC6444425

[55] Jindal G, Sunoj RB. 2012. Revisiting sesquiterpene biosynthetic pathways leading to santalene and its analogues: a comprehensive mechanistic study. Org Biomol Chem 10:7996–8006. doi:10.1039/c2ob26027a. 22951817

[56] Dickschat JS, Brock NL, Citron CA, Tudzynski B. 2011. Biosynthesis of sesquiterpenes by the fungus *Fusarium verticillioides*. Chembiochem 12:2088–2095. doi:10.1002/cbic.201100268. 21748838

[57] Ker DS, Pang SL, Othman NF, Kumaran S, Tan EF, Krishnan T, Chan KG, Othman R, Hassan M, Ng CL. 2017. Purification and biochemical characterization of recombinant *Persicaria* minor β-sesquiphellandrene synthase. Peer J 5:e2961. doi:10.7717/peerj.2961. 28265494 PMC5333544

[58] Beltran-Garcia MJ, Estarron-Espinosa M, Ogura T. 1997. Volatile compounds secreted by the oyster mushroom (*Pleurotus ostreatus*) and their antibacterial activities. J Agric Food Chem 45:4049–4052. doi:10.1021/jf960876i.

[59] Afgan E, Baker D, Batut B, van den Beek M, Bouvier D, Cech M, Chilton J, Clements D, Coraor N, Grüning BA, Guerler A, Hillman-Jackson J, Hiltemann S, Jalili V, Rasche H, Soranzo N, Goecks J, Taylor J, Nekrutenko A, Blankenberg D. 2018. The Galaxy platform for accessible reproducible and collaborative biomedical analyses: 2018 update. Nucleic Acids Res 46:W537–W544. doi:10.1093/nar/gky379. 29790989 PMC6030816

[60] R Core Development Team. 2020. R: a language and environment for statistical computing. R Core Development Team, Vienna, Austria.

[61] Soneson C, Love MI, Robinson MD. 2015. Differential analyses for RNA-seq: transcript-level estimates improve gene-level inferences. F1000Res 4:1521. doi:10.12688/f1000research.7563.2. 26925227 PMC4712774

[62] Love MI, Huber W, Anders S. 2014. Moderated estimation of fold change and dispersion for RNA-seq data with DESeq2. Genome Biol 15:550. doi:10.1186/s13059-014-0550-8. 25516281 PMC4302049

[63] Kolde R. 2019. pheatmap: pretty heatmaps 1.0.12. https://rdrr.io/cran/pheatmap/.

[64] Garnier S, Ross N, Rudis B, Sciaini M, Camargo AP, Scherer C. 2021. Colorblind-friendly-color maps for R. https://cran.r-project.org/web/packages/viridis/vignettes/intro-to-viridis.html

